# Periocular rejuvenation: current concepts and evidence-based management

**DOI:** 10.3389/fmed.2026.1943441

**Published:** 2026-10-12

**Authors:** Alessio Martucci, Flavia Quaranta Leoni, Matteo Di Marino, Enrico Romano

**Affiliations:** 1 Ophthalmology Unit, Department of Experimental Medicine, University of Rome “Tor Vergata”, Rome, Italy; 2 Department of Sense Organs, Sapienza University of Rome, Rome, Italy

**Keywords:** ageing, blepharoplasty, botulinum toxin, collagen biostimulators, dark circles, dermatochalasis, eyelid ageing, hyaluronic acid fillers

## Abstract

The periocular region shows early visible ageing because thin skin, repetitive orbicularis oculi activity, retaining ligaments, orbital fat, and the bony framework change together. Clinical manifestations include rhytids, dermatochalasis, eyelid laxity, tear trough deformity, edema, and dark circles, with potential cosmetic, ocular-surface, and functional consequences. Assessment should distinguish pigmentary, vascular, structural, and volume-related findings and should include eyelid position and laxity, brow position, ocular-surface status, relevant medication use, and visual-field testing when functional symptoms are present. Management is anatomy driven. Botulinum toxin type A, hyaluronic acid fillers, light- and energy-based procedures, and selected regenerative or collagen-biostimulatory injectables may benefit appropriately selected patients, whereas blepharoplasty and fat repositioning remain the reference treatments for substantial tissue laxity, fat prolapse, or functional impairment. Evidence is strongest for established indications but remains heterogeneous across devices, injection techniques, and emerging products. Early positive clinical, objective, mechanistic, and safety data for polynucleotides and collagen biostimulators indicate insufficient evidence to determine comparative efficacy rather than evidence of absent effect. Procedure-specific recommendations should therefore be interpreted according to whether they arise from comparative studies, consensus, anatomical principles, or expert practice.

## 1 Introduction

Periocular ageing affects the skin, eyelids, ocular adnexa, and underlying facial support. It may alter facial expression and produce cosmetic concerns, but it can also contribute to eyelid malposition, ocular-surface symptoms, and visual-field limitation. Progressive age-related changes in the eye and ocular surface may contribute to visual symptoms and, in some cases, visual impairment; lens, retinal, and optic-nerve disease remain major causes of visual loss in older adults. Age-related eyelid changes may result in cosmetic and/or functional concerns and can be addressed when treatment is desired and clinically indicated.

The thin periocular dermis, close relationship to the orbicularis oculi, superficial vasculature, and limited subcutaneous tissue make volume loss, vascular show, pigmentation, and fine rhytids readily visible. Intrinsic ageing, ultraviolet exposure, smoking, and other environmental factors accelerate collagen and elastin fragmentation, pigment dysregulation, and soft-tissue descent (1, 2).

Clinical assessment should identify the predominant mechanism: dynamic muscle activity, skin-quality change, pigment or vascular alteration, volume depletion, ligamentous laxity, orbital fat prolapse, or eyelid malposition. Ethnicity, Fitzpatrick phototype, previous procedures, systemic and ocular comorbidities, relevant medications, and patient expectations influence both treatment choice and risk.

Treatment ranges from topical therapy, botulinum toxin, fillers, resurfacing, radiofrequency, and regenerative or collagen-biostimulatory injectables to blepharoplasty and fat repositioning. Minimally invasive procedures are most useful for selected early or moderate changes; surgery remains the reference treatment for substantial dermatochalasis, fat prolapse, eyelid laxity, or functional impairment (3).

This narrative review integrates periocular anatomy, clinical phenotyping, nonsurgical and surgical treatment, ocular-surface and functional assessment, and management of vision-threatening complications. Its ophthalmology-oriented approach emphasizes when aesthetic findings overlap with ocular disease, when functional testing or referral is required, and how the strength and source of evidence should qualify technical recommendations.

## 2 Material and methods

This narrative review was prepared in accordance with the Scale for the Assessment of Narrative Review Articles (SANRA). PubMed/MEDLINE was searched from database inception through June 2026 using combinations of terms including “periocular rejuvenation”, “eyelid ageing”, “dermatochalasis”, “blepharoplasty”, “tear trough deformity”, “dark circles”, “hyaluronic acid fillers”, “botulinum toxin”, “polynucleotide”, “polydeoxyribonucleotide”, “poly-L-lactic acid”, “poly-D,L-lactic acid”, “polycaprolactone”, and related Medical Subject Headings when applicable. Reference lists were screened manually for additional relevant publications.

The limitations inherent to narrative reviews, including potential publication bias and subjective study selection, were minimized through comprehensive literature screening and preferential inclusion of higher-level evidence whenever available. Preference was given to systematic reviews, randomized controlled trials, prospective cohort studies, consensus statements, and clinically relevant recent publications. Case reports, retrospective series, and expert opinions were included only when higher-level evidence was unavailable or when discussing uncommon complications or emerging therapeutic approaches.

Non-peer-reviewed literature, conference abstracts, and studies lacking adequate methodological information were generally excluded unless they provided unique information regarding rare adverse events or novel techniques. Only articles published in English were considered.

Evidence was appraised qualitatively according to study design, methodological quality, consistency, objective vs. subjective outcomes, duration of follow-up, periocular specificity, and clinical relevance. No formal risk-of-bias or GRADE assessment was performed. We distinguished evidence of limited efficacy from insufficient evidence to establish efficacy or comparative superiority. Technical statements were also identified as arising from comparative clinical evidence, consensus guidance, anatomical rationale, or expert practice.

## 3 Eyelid alterations

Ageing of the ocular adnexa reflects intrinsic biology and cumulative environmental exposure. Loss of tissue elasticity and eyelid tone, ligament attenuation, orbital fat redistribution, and bony remodeling produce dermatochalasis, brow or eyelid ptosis, tear trough deformity, lower-eyelid bags, entropion, or ectropion (4–16).

Collagen and elastic-fiber fragmentation, extracellular-matrix remodeling, and lymphatic change contribute to thin, wrinkled skin, pigment alteration, and edema. In the lower eyelid, septal attenuation, fat pseudoherniation, and midface volume loss accentuate the nasojugal groove and inferior orbital rim; in the upper eyelid, fat-pad displacement and brow descent alter the eyelid contour and may worsen pseudoptosis or superior visual-field obstruction (4, 9, 12–16) (Figure 1, Figure 2).

**Figure 1:**
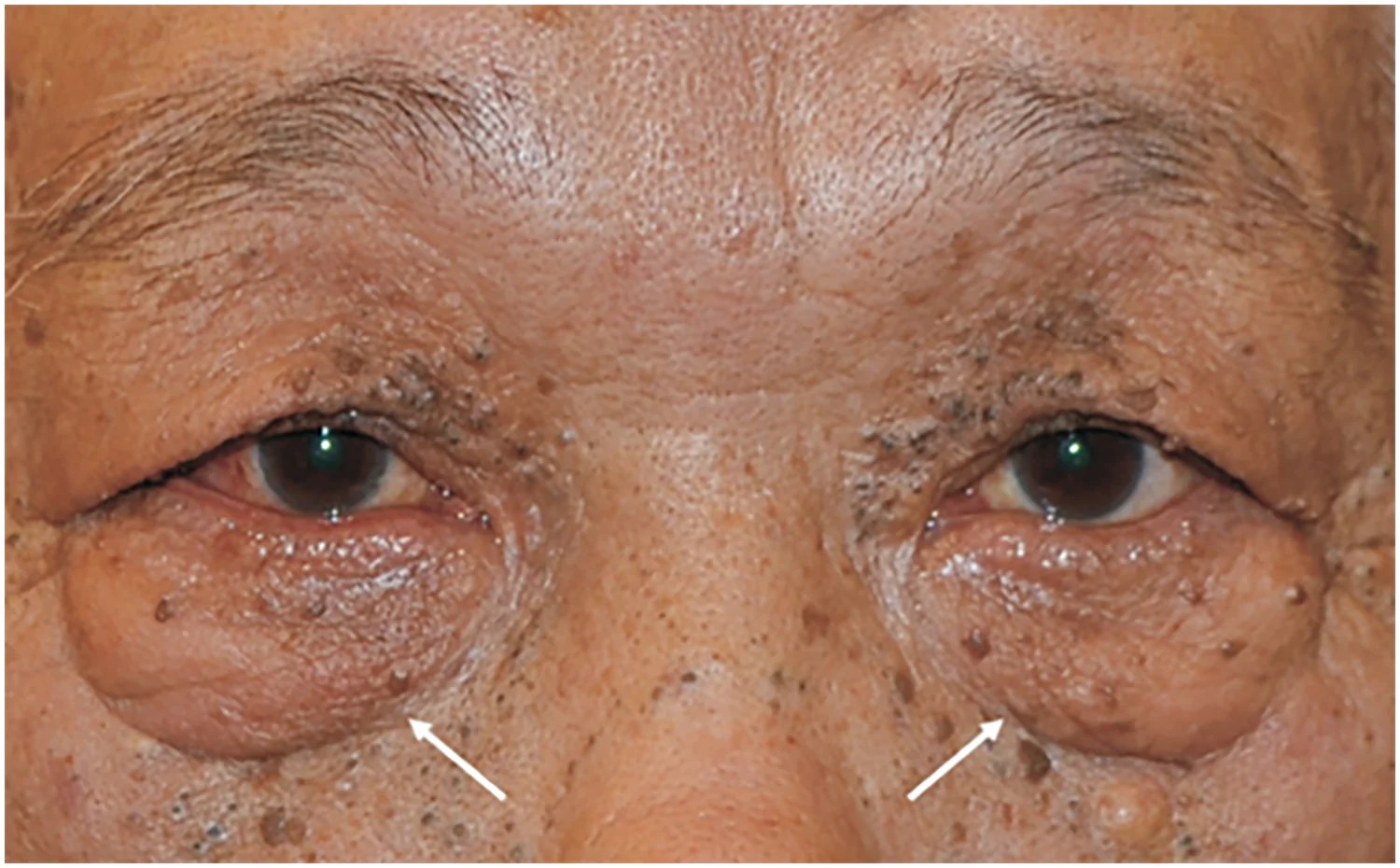
Age-related lower-eyelid change with orbital fat pseudoherniation through an attenuated septum (arrows). Surgical treatment, when indicated, may involve conservative fat reduction or repositioning to improve the lid-cheek contour (16).

**Figure 2:**
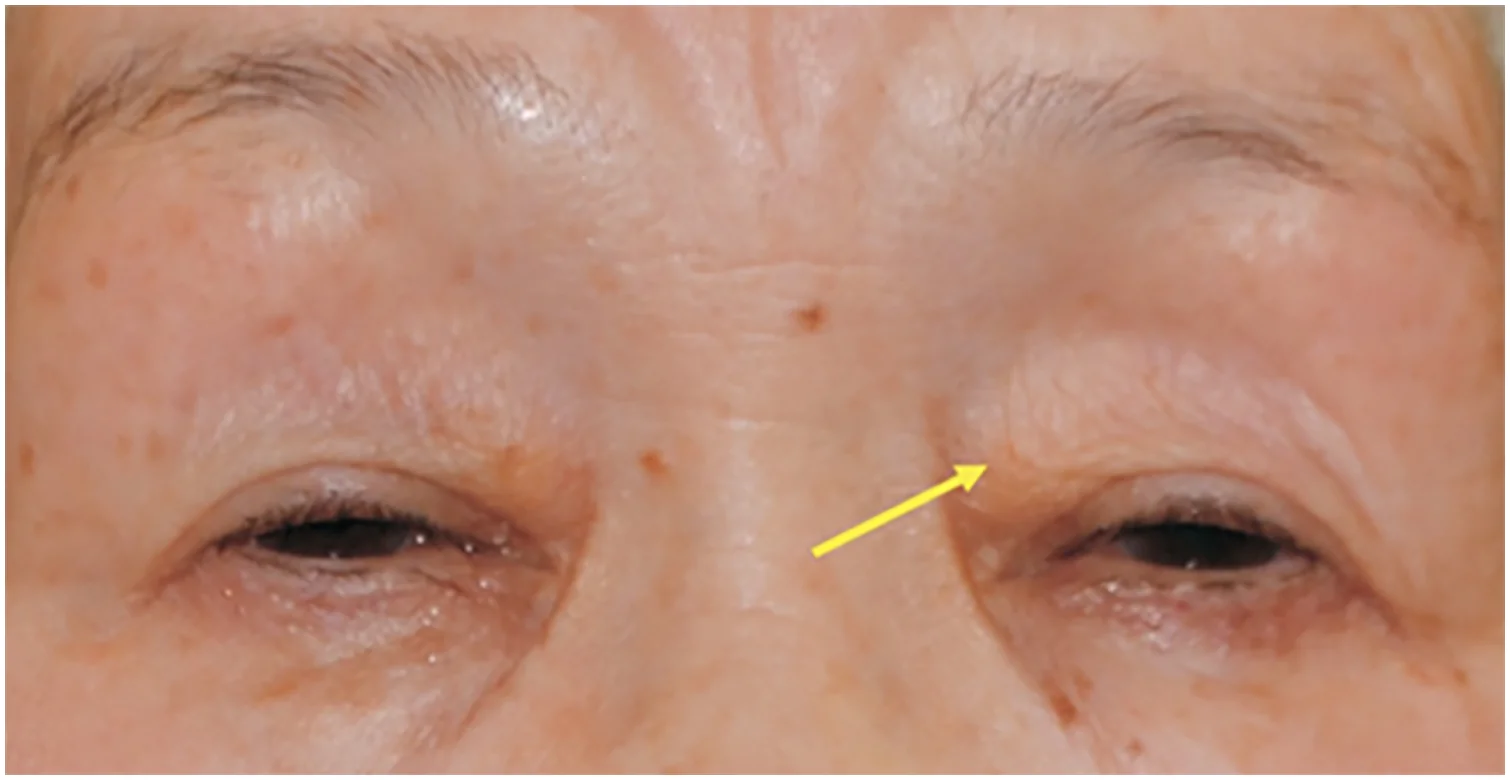
Age-related upper-eyelid change with protrusion of the nasal preaponeurotic fat pad (arrow). Associated dermatochalasis, brow ptosis, and true blepharoptosis should be distinguished during functional assessment (16).

These findings should be recorded as a clinical phenotype before treatment because similar complaints may arise from different mechanisms. Table 1 links common periocular findings to their anatomical contributors, ophthalmic relevance, and key examination steps.

**Table 1:** Principal periocular ageing findings, anatomical contributors, and ophthalmic assessment.

Clinical finding	Principal contributors	Ophthalmic relevance	Key assessment
Dynamic lateral canthal rhytids	Orbicularis oculi activity and dermal ageing	Excessive weakening may affect blink and ocular-surface protection	Rest and animation; blink strength; baseline dry-eye symptoms
Upper-eyelid hooding	Dermatochalasis, brow ptosis, blepharoptosis, or fat prolapse	May obstruct the superior field or prompt compensatory frontalis activity	Brow position, margin-reflex distance 1, levator function, and visual field when indicated
Lower-eyelid laxity or malposition	Canthal tendon laxity, septal change, and orbicularis weakness	Exposure, tearing, keratopathy, ectropion, or entropion	Distraction and snap-back tests; punctal position; corneal surface
Tear trough or lower-eyelid bags	Volume loss, retaining-ligament anatomy, fat pseudoherniation, and midface descent	Thin skin and limited lymphatic reserve increase injection-related edema and contour risk	Tangential illumination, palpation, eyelid support, fat prolapse, and midface volume
Periocular edema	Inflammatory, allergic, thyroid, renal, cardiac, lymphatic, postoperative, or filler-related causes	May signal ocular or systemic disease rather than cosmetic ageing	Onset and fluctuation, systemic review, proptosis, motility, inflammation, and prior filler
Ocular-surface symptoms	Tear-film instability, meibomian dysfunction, exposure, or prior surgery	Procedures may exacerbate dryness, irritation, or fluctuating vision	Symptoms, lid closure, tear film, corneal staining, and referral when abnormal

### 3.1 Ophthalmic assessment and clinical phenotyping

An ophthalmology-oriented assessment extends beyond the visible aesthetic concern. History should address fluctuating or blurred vision, dryness, irritation, contact-lens intolerance, blepharitis or allergy, thyroid disease, previous ocular or eyelid surgery, facial trauma, and medications that alter the periocular tissues, including topical prostaglandin analogues. Baseline standardized photographs should include primary gaze and, when relevant, animation and upgaze.

Examination should document visual acuity when symptoms are present, brow position, margin-reflex distances, levator function, palpebral-fissure height, dermatochalasis, fat prolapse, entropion or ectropion, canthal support, and lower-eyelid distraction and snap-back. Tear-film and corneal-surface evaluation are important before procedures that may worsen exposure or dry eye. Formal visual-field testing is appropriate when upper-eyelid skin or ptosis may cause functional obstruction; proptosis, restrictive motility, inflammation, or unexplained edema warrants investigation before cosmetic treatment (17–25).

Treatment planning should separate skin-quality or contour concerns from ocular-surface disease and eyelid malposition. Functional disease, active infection or inflammation, uncontrolled systemic disease, and unrealistic expectations should be addressed before elective intervention. Laser and light-based procedures require wavelength-appropriate ocular protection, and injections near the orbit require explicit consent regarding rare visual and neurological complications.

### 3.2 Periocular rhytids

Periocular rhytids arise from the interaction of repetitive orbicularis oculi contraction with dermal collagen and elastin loss, photodamage, ligament attenuation, volume loss, and bony remodeling. They usually begin as dynamic lateral canthal lines and may become static as dermal support declines (1–3, 17, 26, 27).

Clinical assessment should distinguish dynamic from static wrinkles and document skin quality, brow position, eyelid laxity, orbicularis activity, lower-eyelid support, tear trough deformity, fat prolapse, and midface volume. Standardized photography at rest and during animation supports treatment planning and follow-up.

Botulinum toxin type A is supported for dynamic rhytids; resurfacing and radiofrequency primarily address texture and dermal remodeling; fillers may benefit selected contour deficits; and blepharoplasty remains the reference treatment for substantial dermatochalasis or tissue laxity. Combined treatment may address coexisting mechanisms, but comparative evidence for specific sequences remains limited (Table 2) (28–54).

**Table 2:** Overview of periocular rejuvenation treatments according to the predominant clinical finding.

Clinical finding	Treatment options	Main rationale	Key considerations	Evidence and basis	References
Dynamic wrinkles and crow's feet	Botulinum toxin A	Reduces orbicularis oculi activity and softens dynamic rhytids	Injection planning should consider periocular muscle balance; possible ptosis, diplopia, and asymmetry	Moderate to high for wrinkle reduction; dosing patterns and some periocular placement details also rely on consensus and expert practice	(29–33, 44–52)
Fine wrinkles and photodamage	Ablative or fractional laser resurfacing	Promotes skin renewal and collagen remodeling	Effective for texture, pigmentation, and rhytids; downtime, pigmentary changes, and ocular protection should be considered	Moderate	(34–39)
Mild periocular laxity	Radiofrequency	Induces dermal heating, collagen contraction, and neocollagenesis	Low downtime and low morbidity; improvement is usually gradual and moderate	Moderate; device-specific protocols remain heterogeneous	(40–43, 55, 56)
Pigmentary dark circles	Topical agents; Q-switched lasers; chemical peels; microneedling with tranexamic acid or vitamin C	Reduce melanin, improve epidermal turnover, and stimulate dermal remodeling	Treatment should be tailored to phototype; irritation and post-inflammatory hyperpigmentation may occur	Low to moderate	(1, 57–81)
Vascular or violaceous dark circles, including vascular-type or mixed dark circles	IPL; pulsed dye laser; carboxytherapy ± adjunctive chemical acids	Targets vascular chromophores and reduces vascular discoloration; carboxytherapy may improve microvascularity	Patient selection according to skin type is important; multiple sessions may be required; evidence for carboxytherapy remains limited	Low to moderate	(1, 55, 75, 76, 82–89)
Tear trough deformity and infraorbital hollowing	HA fillers	Restores volume and improves the lower eyelid–cheek transition	Comparative studies support HA efficacy, but preferred plane, product rheology, aliquot size, and treatment sequence are based mainly on anatomy, consensus, and experienced practice	Moderate for clinical correction; low for comparative procedural technique	(1, 2, 53, 54, 90–109)
Periorbital hyperpigmentation with volume loss or skin-quality impairment	PN or PDRN formulations; PN-HA complexes; PRP; fat-derived therapies; microneedling	Targets skin quality, hydration, pigmentation, and tissue remodeling rather than reliable structural volume replacement	Positive prospective, objective, and mechanistic findings are available, but formulations and protocols vary and comparative superiority is unestablished	Low to moderate; evidence is insufficient to establish comparative efficacy, not evidence of absent clinical effect	(90, 110–119)
Infraorbital hollowing or lower-eyelid skin-quality impairment	PLLA, PDLLA, PDLLA-HA, or liquid PCL collagen biostimulators	Gradual collagen stimulation and tissue remodeling; some products combine biostimulation with HA-associated hydration	Periocular evidence is early; thin skin and restricted lymphatic drainage increase concern for edema, visibility, nodules, and granulomas; non-HA components are not reversed by hyaluronidase	Low and emerging; small retrospective cohorts, prospective series, and pilot studies	(117, 120–124)
HA filler-related complications	Hyaluronidase	Degrades HA and reduces filler-related complications	Specific antidote for HA fillers; careful dosing is required to avoid overcorrection or unintended filler dissolution	Moderate	(103–109, 125–132)
Vision-threatening filler complications	Emergency management protocols; urgent specialist referral; hyaluronidase for suspected HA embolization	No rescue intervention has demonstrated reliable visual recovery after established retinal ischemia; emergency measures aim to avoid delay and may be attempted on a consensus or case-based rationale	Stop injection, activate emergency services, and transfer urgently to an ophthalmic or stroke-capable center; supportive measures must not delay transfer	Very low for visual recovery; emergency pathways are consensus based	(106, 133–141)
Chronic periocular edema	Etiology-directed management	Identifies and treats allergic, inflammatory, thyroid-related, drug-induced, systemic, or lymphatic causes	Underlying disease should be excluded before cosmetic or surgical procedures	Low	(17)
Mild-to-moderate dermatochalasis	Plasma exeresis	Produces tissue sublimation and collagen remodeling, resulting in non-surgical tightening	May be considered when surgery is not desired; not equivalent to blepharoplasty in advanced cases	Low	(27, 131–134, 142–149)
Moderate-to-severe dermatochalasis	Upper blepharoplasty, pinch blepharoplasty, or sub-brow blepharoplasty	Removes redundant skin and may address fat pad prominence or lateral hooding	Appropriate for significant or functional skin excess; planning should consider brow ptosis, dry eye, and asymmetry	Moderate to high	(26, 27, 44, 131, 132, 142, 148–152)
Lower eyelid bags due to fat prolapse	Lower eyelid blepharoplasty ± fat repositioning or septal reset	Reduces or repositions protruding orbital fat and improves lower eyelid contour	Approach depends on skin excess, fat prolapse, eyelid laxity, and tear trough involvement	Moderate	(25, 153–156)

HA, hyaluronic acid; IPL, intense pulsed light; PCL, polycaprolactone; PDLLA, poly-D,L-lactic acid; PLLA, poly-L-lactic acid; PN, polynucleotide; PDRN, polydeoxyribonucleotide. Evidence levels were assigned qualitatively from periocular-specific study design, methodological quality, consistency, objective outcomes, and follow-up. A low rating indicates limited certainty or insufficient comparative evidence and should not be interpreted as proof of absent clinical effect. Technique details may derive from comparative evidence, consensus, anatomical rationale, or expert practice as specified.

#### 3.2.1 Botulinum toxin type A

Botulinum toxin type A (BoNT-A) reduces dynamic lateral canthal rhytids by temporarily inhibiting acetylcholine release and decreasing orbicularis oculi activity. It may also complement blepharoplasty or brow treatment when muscle balance is assessed carefully (31–33).

Randomized trials, prospective studies, and consensus statements support improvement in dynamic periocular wrinkles and high patient satisfaction (44–50). Dosing and injection pattern should reflect anatomy and muscle strength; conservative treatment is common expert practice to preserve expression and limit unwanted diffusion.

Successful treatment requires consideration of the functional balance between the orbicularis oculi, frontalis, glabellar complex, and adjacent facial musculature. Failure to account for these interactions may result in compensatory muscle recruitment, brow asymmetry, or suboptimal aesthetic outcomes (33).

Adverse events are generally mild, transient, and technique dependent. The most frequently reported complications include ecchymosis, headache, temporary eyelid ptosis, brow asymmetry, diplopia, blurred vision, and dry eye symptoms. These events are uncommon when appropriate injection techniques, conservative dosing, and careful patient selection are employed, reinforcing the favorable safety profile of BoNT-A in periocular rejuvenation (44–50).

#### 3.2.2 Hyaluronic acid dermal fillers

Hyaluronic acid (HA) fillers can restore selected periocular volume deficits and improve the lid-cheek transition. Their elasticity, cohesivity, viscosity, and water-binding capacity influence tissue behavior and the risk of edema, migration, contour irregularity, nodules, and the Tyndall effect (1).

Clinical studies support HA correction in appropriately selected patients, but many procedural details are derived from anatomy, consensus, and experienced injector practice rather than comparative trials. Recommendations for conservative volumes, low water-binding products, deep placement, and treatment of midface support before direct tear-trough filling should therefore be interpreted as risk-reduction principles, not universally proven superior techniques (1, 51–54).

Reported benefits may persist for 12–18 months in selected patients, although products, techniques, outcomes, and follow-up differ substantially. Product choice should be individualized rather than inferred from cross-study comparisons, and persistent edema or contour change remains an important periocular limitation (51–54).

### 3.3 Dark circles

Periorbital hyperpigmentation (POH), or dark circles, is a clinical description rather than a single disorder. Pigmentary, vascular, structural, and mixed phenotypes may coexist, and treatment depends on identifying the dominant mechanism (1, 57–71, 82–84, 157–159).

Pigmentary POH appears brown or black because of epidermal or dermal melanin. Vascular POH is blue, pink, or violaceous because thin lower-eyelid skin reveals superficial vessels or congestion. Structural POH results from tear trough deformity, fat pseudoherniation, skin laxity, or midface volume loss that creates shadow rather than true pigment. Constitutional factors, ultraviolet exposure, inflammatory dermatoses, allergy, sleep deprivation, smoking, and ageing can contribute.

Assessment under direct and tangential illumination should document skin color and thickness, vascular prominence, tear trough shape, fat prolapse, laxity, and midface support. Gentle skin stretching helps distinguish persistent pigment from shadowing; standardized photography, colorimetry, melanin or erythema indices, and high-frequency ultrasound can provide objective measures when available (160).

History should include ethnicity and phototype, atopic or contact dermatitis, allergy, systemic disease, previous aesthetic procedures, and ophthalmic medication. Prostaglandin F2alpha analogues may cause reversible periocular pigmentation and, less commonly, prostaglandin-associated periorbitopathy with deepened lid sulci and fat atrophy (18–24).

Management is phenotype directed. Topical depigmenting agents, chemical peels, and pigment-selective lasers address melanin; vascular devices may benefit visible vessels; fillers or surgery address selected structural causes; and regenerative or biostimulatory approaches primarily target skin quality. Mixed phenotypes often require staged multimodal care (Table 2) (1, 51–80, 82–90, 110–116, 161).

#### 3.3.1 Concealers and cosmeceuticals

Topical camouflage and cosmeceutical agents represent the least invasive therapeutic options and may be particularly useful in patients with mild pigmentary dark circles or as adjuncts to procedural treatments. Concealers and color-correcting cosmetics provide immediate improvement by masking discoloration, while optical diffusers containing mineral particles such as boron nitride, mica, silica, and titanium dioxide enhance light reflection and reduce the appearance of infraorbital shadowing (57–60).

Among topical agents, retinoids promote dermal collagen synthesis and epidermal turnover while reducing melanogenesis, thereby improving both pigmentation and skin texture (61). Hydroquinone remains one of the most effective depigmenting agents through inhibition of tyrosinase activity, although its long-term use is limited by potential adverse effects and regulatory restrictions in several countries (62–64). Other depigmenting compounds, including arbutin, kojic acid, tranexamic acid, niacinamide, and vitamin C derivatives, have demonstrated variable efficacy and are frequently incorporated into combination formulations (64, 74). In particular, a novel topical formulation containing niacinamide, arbutin, tranexamic acid, ubiquinone, a DHEA-like component, curcuma extract, and marine exopolysaccharides demonstrated substantial efficacy, achieving an average reduction of nearly 48% in under-eye hyperpigmentation after six weeks of twice-daily application without reported adverse effects.

Topical caffeine may improve vascular congestion and periorbital edema through vasoconstrictive effects, whereas peptides have been proposed to enhance extracellular matrix remodeling by stimulating collagen synthesis and inhibiting collagen degradation (72, 73, 110, 161). Although several topical formulations have demonstrated encouraging clinical results, including combinations containing depigmenting, antioxidant, and anti-inflammatory agents, the available evidence remains heterogeneous, with relatively few high-quality randomized controlled trials directly comparing different products (74).

#### 3.3.2 Intense pulsed light and phototype considerations

Intense pulsed light (IPL) targets melanin and hemoglobin and may benefit vascular or mixed POH. Parameters should be selected according to the target chromophore and Fitzpatrick phototype. Types III–VI have greater risk of post-inflammatory hyperpigmentation after IPL, pulsed dye laser, and chemical peeling; recent tanning, active inflammation, inadequate photoprotection, and aggressive settings increase that risk. Conservative parameters, strict ocular protection, pre- and post-treatment photoprotection, and, in selected patients, pigment-suppressing regimens are practical risk-reduction measures (36, 75, 76, 162).

#### 3.3.3 Radiofrequency

Radiofrequency (RF) devices use electric current-generated energy to produce a thermal effect that induces collagen contraction and neocollagenesis while minimizing collateral damage. Unlike light-based technologies, RF energy is not affected by tissue absorption or scattering, allowing treatment of deeper layers such as dermis and subcutaneous fat without causing thermal burns, however, tissue heating is influenced by electrical impedance, device configuration, energy delivery, and contact conditions, and burns or ocular injury remain possible (76).

RF can be used across a wide range of skin types and has been applied for periorbital skin tightening. The resulting collagen contraction is generally mild to moderate and more subtle than that achieved with ablative or fractionated lasers, but the low morbidity makes RF a favorable option when modest improvements with minimal risk are desired (1, 40–43).

Sublative fractional radiofrequency has emerged as a promising non-invasive treatment option for periorbital hyperpigmentation. In a prospective study, SFR treatment resulted in significant improvements in periorbital skin lightness, elasticity, and overall skin quality, accompanied by a reduction in melanin content and visible improvement in pigmentation and wrinkles. Objective assessments demonstrated increased epidermal and dermal density, suggesting tissue remodeling and skin rejuvenation effects. Both physician evaluations and patient-reported outcomes confirmed high levels of treatment satisfaction, while no serious adverse events were observed. These findings indicate that sublative fractional radiofrequency is a safe, effective, and well-tolerated modality for managing periorbital hyperpigmentation, offering benefits that extend beyond pigmentation reduction to include improvements in skin texture and signs of periorbital ageing (85).

#### 3.3.4 Laser treatment

Q-switched lasers are effective for treating dark circles caused by hyperpigmentation, as their nanosecond pulse technology allows selective targeting of melanosomes with minimal damage to surrounding tissues. Ruby Q-switched lasers (694 nm) disrupt melanosomes in melanocytes, melanophages, and keratinocytes, making them suitable for Fitzpatrick types I and II, though less ideal for darker skin. Studies report over 40% improvement in pigmented dark circles with this modality (65).

Adjunctive use of retinoids and hydroquinone can enhance safety and address residual hyperpigmentation (66).

Alexandrite Q-switched lasers (755 nm) provide greater safety for higher Fitzpatrick types due to reduced melanosome disruption, while Nd:YAG lasers penetrate deeper with minimal melanosome effect, making them suitable for darker skin types (V–VI) (1, 67–69).

Pulsed dye laser (PDL) therapy selectively targets hemoglobin and is therefore most appropriate for vascular dark circles characterized by prominent superficial vasculature or erythematous discoloration. They emit light at 585–595 nm with pulse durations of 0.45–40 ms and are best suited for Fitzpatrick I–III skin types. Treatments are typically performed every 4–6 weeks and require multiple sessions. Current evidence supports its efficacy in selected vascular lesions, although data specifically addressing infraorbital dark circles remain relatively limited, and treatment should be reserved for carefully selected patients (1, 82–84).

Ablative resurfacing improves infraorbital dark circles by treating pigment irregularities, enhancing skin tightness, and increasing collagen and ECM, thereby improving camouflage of underlying orbicularis muscle and vasculature. The mechanism involves controlled skin injury followed by regeneration with increased collagen, reduced pigmentation, and fewer rhytids.

CO₂ and erbium:YAG (Er:YAG) lasers are the main non-fractionated technologies. CO₂ lasers target water with deeper penetration and greater thermal damage, while Er:YAG lasers provide more precise superficial ablation with minimal thermal injury (34, 35).

Fractionated laser resurfacing delivers similar benefits with a pixelated pattern of treatment, leaving areas of intact skin to reduce downtime and complications. Outcomes are comparable to traditional ablative methods but with improved safety and recovery profiles (1, 36–39) (Figures 3A–D).

**Figure 3:**
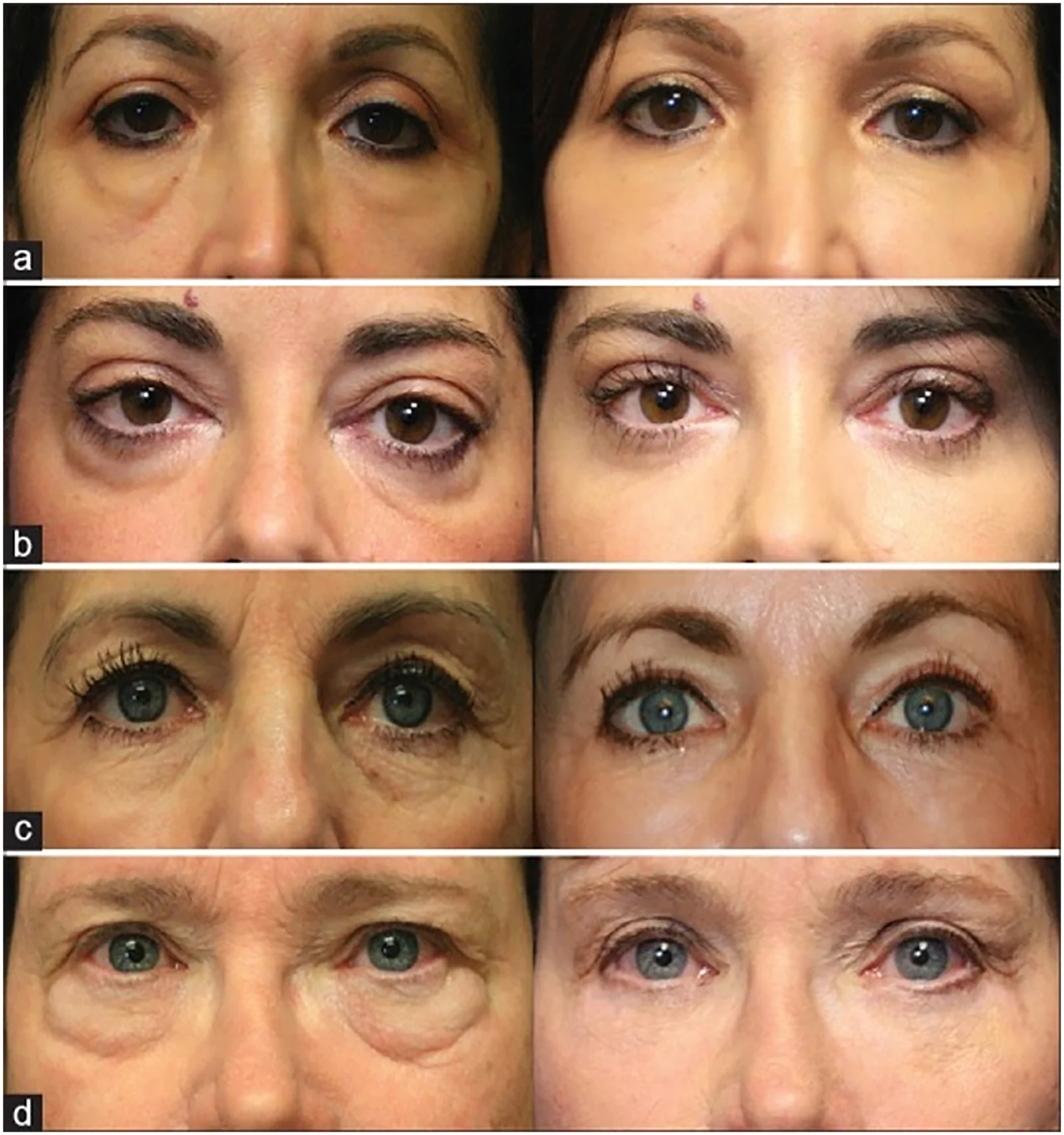
Before-and-after examples of infraorbital dark-circle treatment: HA gel volume enhancement **(a)**, autologous fat transfer **(b)**, laser resurfacing **(c)**, and lower blepharoplasty with orbital fat transposition **(d)** (1).

#### 3.3.5 Chemical peel

Chemical peeling can be used alone or combined with other treatments for dark circles, addressing both pigment irregularities and rhytids. It also improves collagen, helping to mask underlying orbicularis muscle and vasculature.

Chemical peels represent an effective treatment option for pigmentary forms of periorbital hyperpigmentation (POH), particularly constitutional dark circles and periorbital melanosis. Deep phenol–croton oil peels have demonstrated substantial and durable clinical improvement, with most patients achieving greater than 50% reduction in pigmentation and sustained benefits lasting up to several years, while maintaining a low complication rate (55).

Superficial and medium-depth peels, including lactic acid, mandelic acid, and phenol–trichloroacetic acid (TCA) combinations, have also shown significant efficacy in reducing pigmentation and improving cosmetic appearance, with favorable safety profiles and high patient satisfaction (56, 77, 78). Trichloroacetic acid (TCA) peels, available in varying concentrations, remove melanin from superficial to deeper layers depending on depth. They are effective particularly in Fitzpatrick I–III skin types, while use in more pigmented skin requires caution due to risk of pigmentary complications (70).

Studies using combined 3.75% TCA and 15% lactic acid have shown improvement in over 90% of patients with Fitzpatrick II–IV skin types (1, 71).

Comparative studies suggest that lactic acid peels may provide greater pigment reduction and patient satisfaction than mandelic acid peels. A systematic review further supports the role of chemical peels as valuable treatment modalities for pigment-based POH, while emphasizing that treatment selection should be guided by the underlying etiology of dark circles. Overall, current evidence indicates that chemical peeling is a safe and effective strategy for selected patients with periorbital hyperpigmentation, although larger randomized controlled studies are needed to strengthen the evidence base and optimize treatment protocols (56, 77, 78).

#### 3.3.6 Carboxytherapy

Carboxytherapy delivers medical-grade carbon dioxide and has been studied for vascular or mixed periorbital dark circles. Small clinical studies report reduced severity, improved microvascular imaging findings, extracellular-matrix change, and benefits lasting up to six months, including when combined with selected acids (79, 80, 86, 87, 89). These results constitute positive preliminary clinical evidence. Small samples, nonrandomized designs, heterogeneous protocols, and limited controls nevertheless prevent firm conclusions about causality, durability, or comparative efficacy.

#### 3.3.7 Injectables

Injectable approaches address different components of dark circles. HA and autologous fat primarily correct volume-related shadowing; collagen products, PN formulations, PRP, cell-free fat extract, and nanofat are intended to improve skin quality, pigmentation, or remodeling. Small comparative, prospective, and objective studies report clinical improvement, but products and outcomes are too heterogeneous to infer class-wide efficacy or superiority (88–90, 110–116, 161).

The available evidence should therefore be described as limited or insufficient for comparative conclusions, not as showing absent clinical effect. Structural correction, skin-quality treatment, and pigment treatment should be selected according to the dominant phenotype, with explicit discussion of the maturity of evidence for each product.

### 3.4 Tear trough deformity

Tear trough deformity is a depression along the infraorbital rim caused by variable contributions from inherited anatomy, midface and fat-compartment volume loss, orbicularis retaining-ligament attenuation, skin thinning, maxillary remodeling, and orbital fat pseudoherniation. It often coexists with dark circles, lower-eyelid laxity, dermatochalasis, and malar descent (163–168).

Evaluation should include standardized lighting, tangential illumination, skin-stretch testing, lower-eyelid support, distraction and snap-back tests, fat prolapse, and midface volume. Classification systems can aid communication, but none establishes a universal treatment sequence (163, 169, 170).

Treatment should address the dominant anatomy. HA filler may improve localized volume deficiency in selected patients; marked skin excess, fat prolapse, or eyelid laxity favors surgical treatment. Midface correction may reduce the apparent trough without direct infraorbital injection, but this sequence is supported mainly by anatomical rationale and expert consensus rather than definitive comparative trials (25, 153, 154, 163–173).

#### 3.4.1 Lower eyelid blepharoplasty

Lower-eyelid blepharoplasty may conservatively reduce, preserve, or reposition protruding orbital fat according to the patient's anatomy (156). A transcutaneous approach permits treatment of skin and muscle but carries a greater risk of retraction or ectropion unless lower-eyelid support is addressed; a transconjunctival approach avoids an external skin incision but may be followed by transient chemosis, irritation, or dry-eye symptoms (25, 156) (Figures 4a,b). Fat repositioning or septal reset can improve a persistent trough while limiting postoperative hollowing (155).

**Figure 4:**
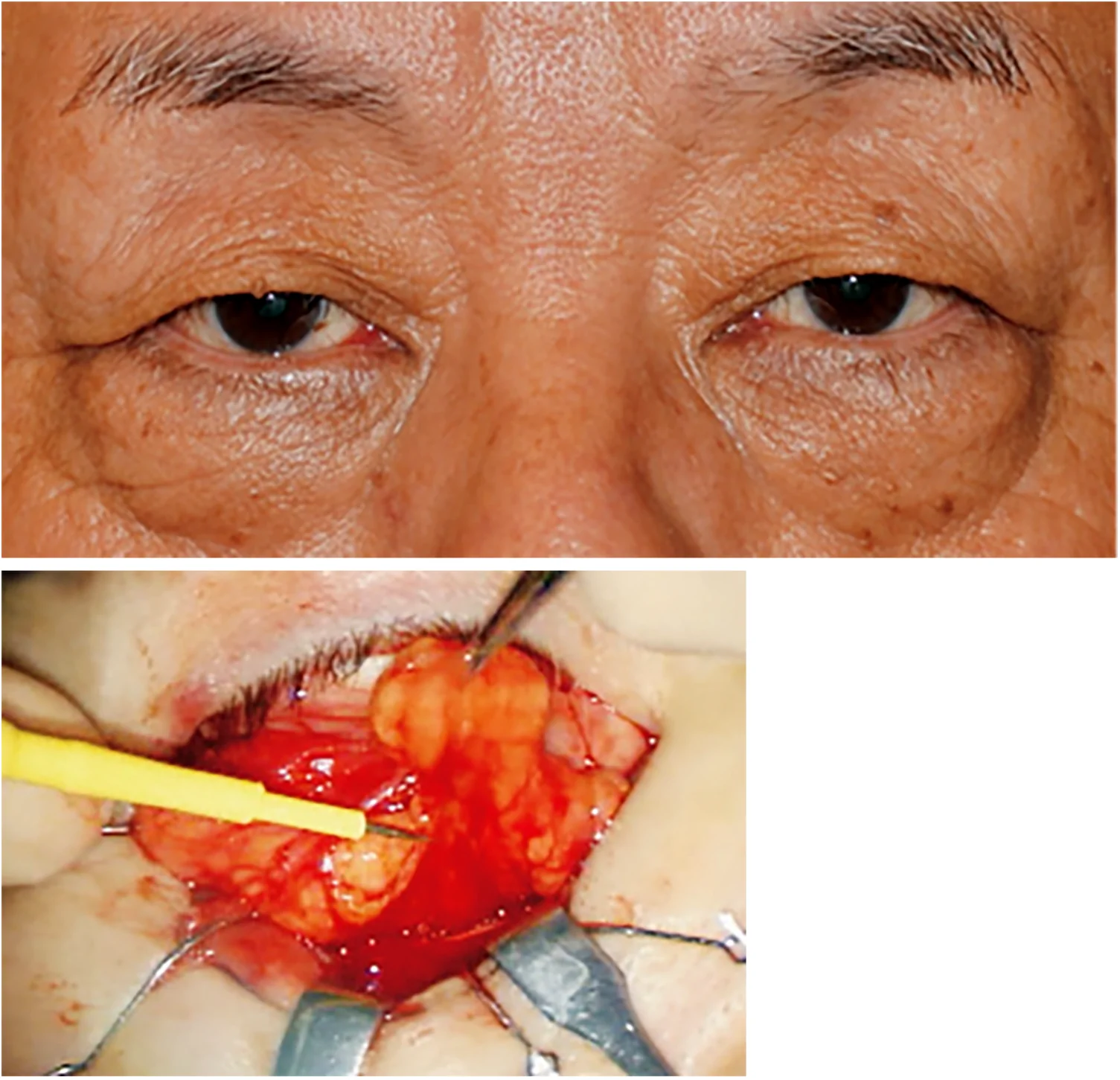
**(a)** lower-eyelid bags before treatment (16). **(b)** Intraoperative lower-eyelid blepharoplasty (16).

#### 3.4.2 Hyaluronic acid injectables

HA fillers are used for selected tear trough and lid-cheek contour deficits because correction is immediate and HA can be dissolved with hyaluronidase. Several assessment systems distinguish direct correction from an indirect approach through adjacent cheek or temple support, but their treatment algorithms are based primarily on anatomy and expert practice rather than head-to-head trials (2, 91–98).

Systematic reviews and prospective studies support clinical improvement after HA treatment. Deep preperiosteal or pre-periosteal placement, low water-binding products, and small aliquots are common consensus-based risk-reduction strategies in the thin lower eyelid. Suggested initial ranges such as 0.1–0.7 mL per side represent experienced practice, not a validated universal maximum (81, 92–100).

Evidence does not show that either a needle or cannula is universally safer or more effective. Comparative data suggest less bruising or vascular trauma with cannulas in some settings and greater placement precision with needles, with similar longer-term satisfaction. Device choice should therefore depend on anatomy and operator expertise rather than an assumed categorical safety advantage (100, 101). Delayed edema, contour irregularity, Tyndall effect, nodules, and rare vascular occlusion should be included in consent.

#### 3.4.3 Regenerative and collagen biostimulatory injectables

Polynucleotides (PN) and polydeoxyribonucleotide (PDRN) are related DNA-derived products but should not be treated as interchangeable labels. PN products generally contain longer polymer chains intended to support hydration and extracellular-matrix remodeling, whereas lower-molecular-weight PDRN is discussed mainly in relation to adenosine A2A-receptor signaling and nucleotide salvage pathways. Periocular PN studies include objective imaging, randomized split-face data, real-world cohorts, and early PN-HA clinical observations; a systematic review found promising improvements in wrinkles, texture, and elasticity but low-to-moderate study quality and heterogeneous protocols (110, 116, 118, 119). PDRN-specific periocular evidence is less developed, so mechanistic and broader skin-repair findings should not be assumed to prove comparative periocular efficacy.

Collagen biostimulators form a separate emerging category. PLLA, PDLLA, PDLLA-HA, and PCL aim to produce gradual fibroplasia and tissue remodeling rather than the immediate, readily reversible correction of HA alone. A retrospective series of 28 PLLA-treated patients reported improvement in infraorbital hollowing with bruising, swelling, and one palpable nodule; small PDLLA and prospective PDLLA-HA cohorts reported improvement in lower-eyelid contour and skin-quality measures; and a three-patient liquid PCL pilot reported short-term structural and functional changes (121–124). These studies demonstrate clinical evidence but do not establish superiority, optimal protocols, or long-term safety.

Periocular-specific caution is required because thin translucent skin, restricted lymphatic drainage, and proximity to the orbit increase the consequences of edema, product visibility, irregularity, nodules, granulomas, and vascular occlusion. Unlike HA, PLLA, PDLLA, and PCL are not reversed by hyaluronidase; in hybrid products, hyaluronidase addresses only the HA component. Use should therefore be limited to carefully selected patients and experienced clinicians, with conservative technique, explicit consent, and follow-up appropriate to an emerging evidence base.

### 3.5 Periocular edema

Thin eyelid skin, minimal subcutaneous tissue, complex venous and lymphatic drainage, and the orbital and malar septa make small fluid shifts clinically visible. True edema should be distinguished from fat pseudoherniation, malar mounds, festoons, filler contour irregularity, and soft-tissue descent (1, 17, 126–130).

Causes include allergy, blepharitis, thyroid eye disease, renal or cardiac disease, lymphatic dysfunction, surgery, and aesthetic treatment. Following tear trough HA injection, superficial placement, excess volume, product water binding, impaired lymphatic drainage, or inflammatory reaction may produce persistent swelling (103–109, 125–130).

History and examination should establish timing, fluctuation, laterality, inflammation, systemic symptoms, previous filler or surgery, eyelid laxity, proptosis, motility, ocular-surface findings, and signs of infection or vascular compromise. Persistent filler-associated edema must be distinguished from delayed inflammatory reaction, granuloma, and infection because management differs.

Treatment is cause specific. Mild postoperative edema often responds to head elevation, cold compresses, and observation. HA-related edema can improve after localized hyaluronidase, sometimes requiring more than one session; dosing should be individualized to avoid unnecessary dissolution or contour loss (106–109, 130) (Table 2).

Festoons and chronic malar edema may respond poorly to conservative measures. Selected patients may require lower-eyelid surgery, septal modification, or preseptal orbicularis treatment, while inflammatory or systemic disease requires appropriate medical or multidisciplinary care (17).

### 3.6 Dermatochalasis

Dermatochalasis is redundant upper- or lower-eyelid skin associated with dermal elastin and collagen loss, connective-tissue and septal attenuation, ultraviolet exposure, inflammation, and gravity. Brow ptosis, true blepharoptosis, fat prolapse, and lymphatic change may coexist and should be assessed separately (9, 12, 27).

Patients commonly present with upper eyelid heaviness, redundant skin folds, visual fatigue, and cosmetic concerns. (Figure 5a) Advanced cases may produce greater visual field impairment, compensatory frontalis muscle activation, brow ache, and difficulty with reading or prolonged visual tasks. Lower eyelid dermatochalasis frequently coexists with orbital fat prolapse, tear trough deformity, and skin rhytids, contributing to an aged appearance (16).

**Figure 5:**
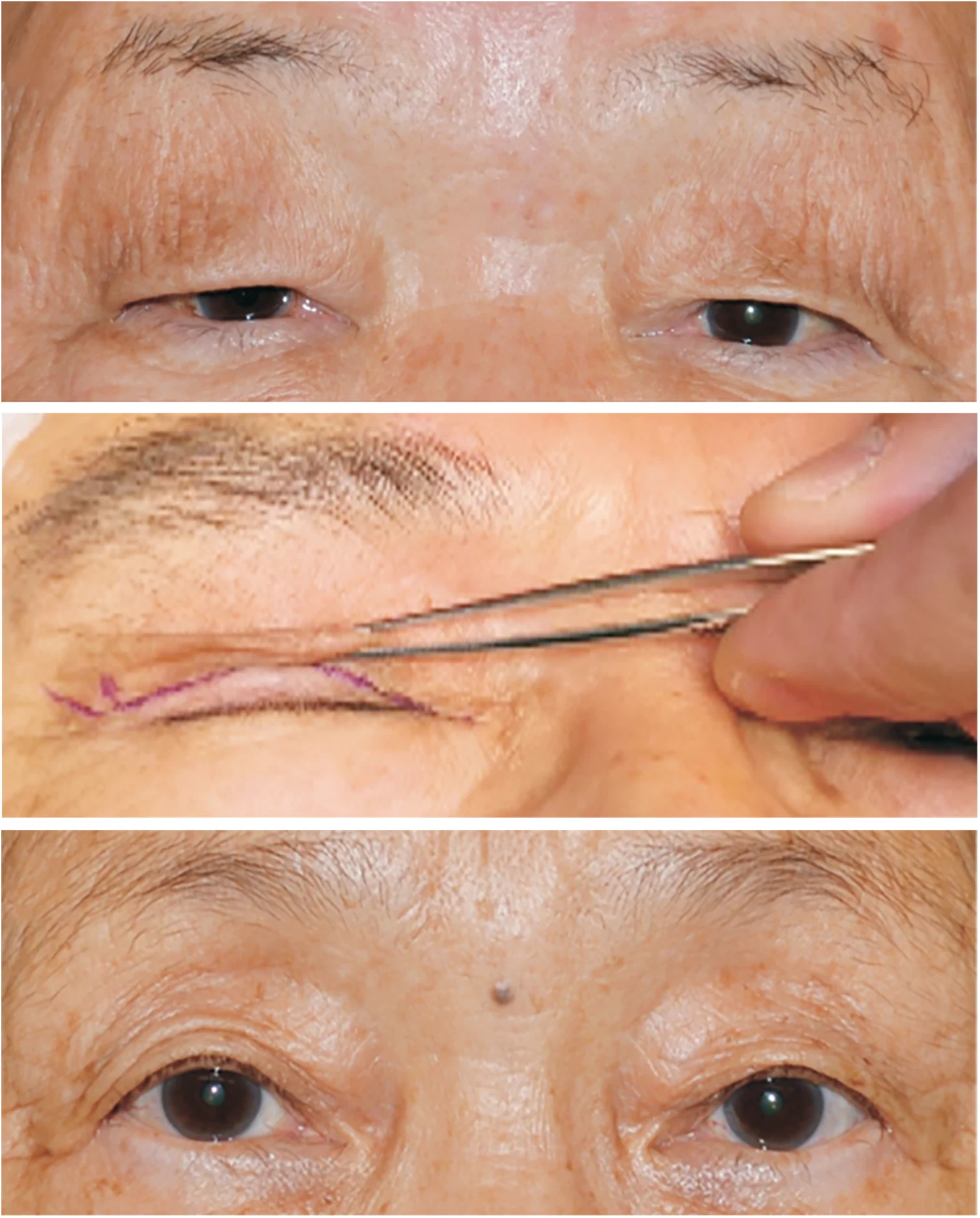
**(a)** upper-eyelid dermatochalasis before pinch blepharoplasty (16). **(b)** Intraoperative marking and skin pinching during upper blepharoplasty (16). **(c)** Postoperative outcome after pinch blepharoplasty (16).

Diagnosis is primarily clinical and should include assessment of eyelid skin redundancy, brow position, levator function, eyelid margin position, fat prolapse, ocular surface status, and visual field impairment when functional symptoms are present. Standardized photography is useful for documentation and treatment planning. Differentiation from brow ptosis, blepharoptosis, eyelid edema, and other causes of pseudo-dermatochalasis is essential to guide appropriate management.

Standardized photography is useful for documentation and treatment planning. Differentiation from brow ptosis, blepharoptosis, eyelid edema, and other causes of pseudo-dermatochalasis is essential to guide appropriate management.

Treatment should be individualized according to the severity of tissue laxity, associated anatomical abnormalities, and patient expectations. Mild cases may benefit from minimally invasive approaches including botulinum toxin, injectable fillers, energy-based devices, or plasma exeresis in selected patients. However, upper or lower blepharoplasty remains the gold standard for moderate-to-severe dermatochalasis, providing durable functional and aesthetic improvement. Surgical planning should address associated brow ptosis, fat prolapse, and tear trough deformity whenever indicated to achieve harmonious periocular rejuvenation. Treatment involves pinch blepharoplasty, where excess skin is excised and prominent nasal fat pads may be reduced, while central fat is usually preserved (Figures 5b,c). In East Asian patients, sub-brow blepharoplasty is often preferred due to eyelid anatomy and lateral hooding. (Figures 6A–C) (16, 27).

**Figure 6:**
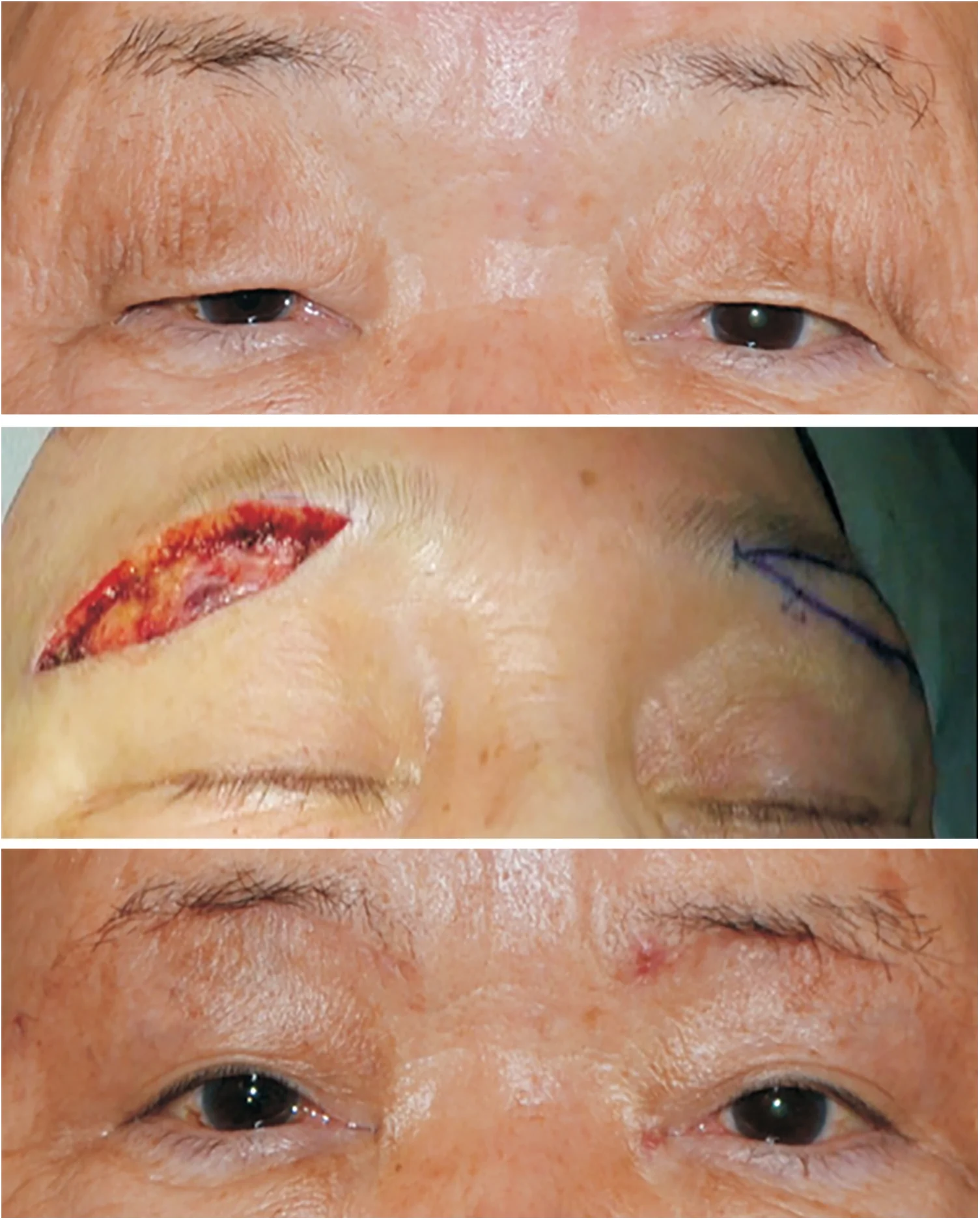
**(a)** dermatochalasis before sub-brow blepharoplasty (16). **(b)** Intraoperative sub-brow blepharoplasty (16). **(c)** Postoperative outcome after sub-brow blepharoplasty (16).

#### 3.6.1 Plasma exeresis

Plasma exeresis produces superficial tissue sublimation and thermal contraction and has been proposed for selected patients with mild-to-moderate dermatochalasis who decline surgery. Small clinical, imaging, and histological studies report improved laxity and findings consistent with collagen remodeling (142–145). The procedure typically involves topical anesthesia followed by precise application of plasma energy over multiple treatment sessions, with post-procedural care focused on hygiene measures, photoprotection, and support of natural healing processes (143).

Evidence is derived mainly from small observational or noncomparative studies with short follow-up and subjective outcomes. Plasma exeresis should not be considered equivalent to blepharoplasty for advanced or functional skin excess, and comparative efficacy, durability, patient selection, and long-term safety remain insufficiently established (146, 147) (Table 2).

## 4 Complications of periocular procedures

Periocular aesthetic procedures can cause transient dry-eye symptoms, irritation, edema, ecchymosis, ptosis, diplopia, or eyelid malposition.

Rare intravascular filler injection can cause retinal or ophthalmic artery occlusion, ischemic optic neuropathy, stroke, and permanent blindness because facial arterial branches communicate with the ophthalmic circulation (133–137).

Visual prognosis after established retinal ischemia is poor. In the American Academy of Ophthalmology review, reported treatments were usually ineffective; final vision was unchanged in 70% of described cases (137). This limitation must be stated before emergency measures are listed: ocular massage, intraocular-pressure reduction, aspirin, hyaluronidase, hyperbaric oxygen, and invasive procedures have not demonstrated reliable restoration of vision after established occlusion.

The absence of a proven rescue does not justify inaction. Because injury is time critical and no superior alternative exists, emergency measures supported by biological plausibility, case reports, or consensus may be started while urgent transfer is arranged, provided that they do not delay access to ophthalmic and stroke-capable care (138–141).

### 4.1 Intravascular filler injection: prevention, recognition and emergency response

Preparedness should include informed consent for rare vision loss and stroke, training to recognize vascular occlusion, an identified emergency department and ophthalmic or stroke pathway, accessible contact information, and ready availability of hyaluronidase for HA-related complications (138–140).

If sudden visual loss, ocular pain, ophthalmoplegia, ptosis, skin pallor or livedoid change, headache, aphasia, weakness, or another stroke-like symptom occurs during or after injection, the procedure should stop immediately.

Emergency services should be activated and the patient transferred without delay to a center with emergency ophthalmology and neurological or stroke capability.

Evidence and consensus-based protocols to help injectors manage vision-threatening complications of filler embolism, and the preliminary guidelines are delineated below (138, 139):

1. Preparation:
  1.1. Have a direct contact with a local stroke team and any sub-specialists who can be readily available, with signs posted and staff trained.
  1.2. Share a protocol/paper for intra-arterial filler dissolution with the stroke team.
  1.3. Keep a minimum of 1500U of hyaluronidase in the office at all times with a plan of how to urgently access more as needed.
  1.4. Obtain a baseline visual acuity and photos.
2. Recognise ischemia by pain or pallor (no epinephrine in any anaesthetic block if used)
3. Immediately stop injecting and plan to treat with hyaluronidase as described below, repeating injections every 15 min.
4. Have a team member immediately call emergency services (e.g., 911) to activate the stroke team and sub-specialists for urgent transfer to a stroke hospital. While the patient is being prepared for transfer, initiate treatment with ocular massage and hyaluronidase as below and accompany the patient during the transfer to continue the treatment en route to the stroke hospital.
5. Initiate ocular massage and instruct the patient to continue the massage with circular, continuous pressure over the closed eye, cycling between massaging for 5 s and resting for 5 s
6. Flood the supraorbital/supratrochlear region with hyaluronidase with 600–1600 U (attempting intra-arterial administration if possible through the supraorbital/ supratrochlear foramina/notches). Percutaneous intra-arterial hyaluronidase through the facial, supraorbital, supratrochlear or angular arteries can also be attempted. Peribulbar or retrobulbar administration of hyaluronidase can be considered by an ophthalmologist as well if there is no additional risk; there is no clear evidence that the hyaluronidase can cross the blood-brain barrier into the central retinal artery.
7. Consider adjuvant treatments including oral aspirin, intraocular pressure-lowering agents or anterior chamber paracentesis, IV or oral steroids, hyperbaric oxygen, and vasodilators such as phosphodiesterase inhibitors (e.g., sildenafil) (138).

Similarly, in 2026 Final Consensus Statements Reached by Multidisciplinary Steering Group stated that:

1. Aesthetic clinicians performing soft tissue filler injections should know their local emergency department (ED) location, and are highly recommended to have this printed out and accessible (name, address, etc). This is to reduce confusion and delay of transfer in the event of an emergency. We would also recommend that aesthetic clinicians consider telephoning the ED when en route to prepare for arrival, although this would need to be discussed by individuals with the relevant stakeholders at a local level.
2. Patients receiving tissue filler should be consented for the risk of vision loss and/or stroke, with the risk described as “rare.”
3. In the event of a possible vascular occlusion following tissue filler with evidence or suspicion of ophthalmic or cerebrovascular involvement, patients should be taken to the closest ED, without delay, so that they can be formally assessed for these possibilities.
4. Best practice would be for the aesthetic clinician to accompany the patient to the ED and to bring with them hyaluronidase (at least 7,500 IU). This would likely result in the patient, who is likely to be significantly distressed, arriving more quickly.
5. In the event of suspected filler-induced vision loss, the following interventions are recommended, although these interventions should not significantly delay transfer to ED.
  (1) Ocular massage
  (2) Topical timolol eye drops, 1 drop
  (3) 300-mg aspirin, single immediate dose (if no contraindication, e.g., recent hemorrhagic stroke)
  (4) Rebreathing in a paper bag
6. There is some evidence that periocular hyaluronidase injection may be beneficial for the visual prognosis, and so it would be reasonable for the aesthetic clinician to attempt this if filler-induced vision loss is suspected, and if they are confident performing this procedure.
7. It is recommended that all aesthetic clinicians performing tissue filler injections attend a course on safety that includes best practice management in the event of a presumed vascular adverse event (139).

More invasive procedures, such as anterior chamber paracentesis, should be performed only by ophthalmologists. Retrobulbar or peribulbar hyaluronidase may be considered by ophthalmologists in selected cases, although its efficacy in established retinal artery occlusion remains limited and controversial. These interventions should not be attempted by non-specialists and should never delay urgent transfer to emergency ophthalmic care (138, 139).

## 5 Discussion and conclusions

Periocular rejuvenation has evolved substantially over recent decades, with increasing emphasis on minimally invasive and tissue-preserving approaches that aim to restore anatomy and function while minimizing morbidity. Contemporary management has shifted from traditional “one-size-fits-all” paradigms toward individualized treatment strategies tailored to each patient's unique anatomical characteristics, ageing pattern, and aesthetic goals. Concurrently, office-based aesthetic procedures have gained widespread acceptance, supported by a growing body of evidence demonstrating the safety and efficacy of injectable agents and other nonsurgical modalities. Nevertheless, the periocular region remains one of the most anatomically complex and technically demanding areas to treat, requiring a thorough understanding of regional anatomy, age-related changes, and potential complications.

Periocular rejuvenation is best organized by clinical phenotype and ophthalmic risk rather than by procedure. Dynamic rhytids, pigmentary or vascular change, structural hollowing, fat prolapse, dermatochalasis, eyelid malposition, edema, and ocular-surface disease require different assessments and treatments. The ophthalmic examination is especially important when symptoms, eyelid laxity, ptosis, proptosis, prior surgery, or medication-related periorbital change may alter diagnosis or safety.

Evidence is strongest for BoNT-A in dynamic lateral canthal rhytids, selected laser indications, HA filler for carefully selected volume deficits, and blepharoplasty for substantial dermatochalasis or fat prolapse. Even for established treatments, specific recommendations about device settings, product rheology, injection plane, needle or cannula choice, volume, and sequence often rest on consensus, anatomical rationale, or expert practice. They should not be presented with greater certainty than the supporting evidence allows.

PN, PDRN, PRP, skin boosters, and collagen biostimulators have positive mechanistic and early clinical data, including objective and prospective periocular studies. The principal limitation is insufficient standardized comparative and long-term evidence, not evidence that these interventions lack clinical activity. Recent PLLA, PDLLA, PDLLA-HA, and liquid PCL reports expand the clinical literature but remain small retrospective cohorts, prospective series, or pilot studies (117–124).

Future studies should use validated ophthalmic and aesthetic outcomes, standardized photography and device parameters, transparent adverse-event reporting, longer follow-up, and direct comparisons. For filler-induced visual loss, prevention and immediate transfer remain central because no rescue intervention reliably restores vision after established retinal ischemia. This evidence-calibrated, ophthalmology-oriented framework supports treatment that addresses patient goals without separating aesthetic outcome from eyelid function, ocular-surface health, and vision safety.
